# Blue rubber bleb nevus syndrome and multiple glomangiomas: report of two cases highlighting the importance of the histological analysis^☆^

**DOI:** 10.1016/j.abd.2022.04.013

**Published:** 2023-02-17

**Authors:** Elsa Stella Mosquera-Belalcazar, Aline Alves Domingues, Alessandra Coppini, Laura Luzzatto, Ana Elisa Kiszewski

**Affiliations:** Service of Dermatology, Santa Casa de Misericórdia de Porto Alegre, Universidade Federal de Ciências da Saúde de Porto Alegre, Porto Alegre, RS, Brazil; Department of Pathology, Santa Casa de Misericórdia de Porto Alegre, Porto Alegre, RS, Brazil; a Service of Dermatology, Santa Casa de Misericórdia de Porto Alegre, Universidade Federal de Ciências da Saúde de Porto Alegre, Porto Alegre, RS, Brazil; b Department of Internal Medicine, Universidade Federal de Ciências da Saúde de Porto Alegre, Porto Alegre, RS, Brazil; c Pediatric Dermatology Unit, Dermatology Service, Santa Casa de Misericórdia de Porto Alegre, Universidade Federal de Ciências da Saúde de Porto Alegre, Porto Alegre, Brazil

Dear Editor,

Blue rubber bleb nevus syndrome (BRBNS) is characterized by multiple venous malformations that often affect the skin and gastrointestinal tract, in some cases causing intestinal bleeding and iron-deficiency anemia.^1^ BRBNS is associated with an autosomal dominant inheritance and equally affects males and females.^2^ Glomangiomas arise from modified perivascular muscle cells, more often affect the male sex, and have an autosomal dominant inheritance pattern.^3^

## Case reports

### Case 1

**Figure 1:**
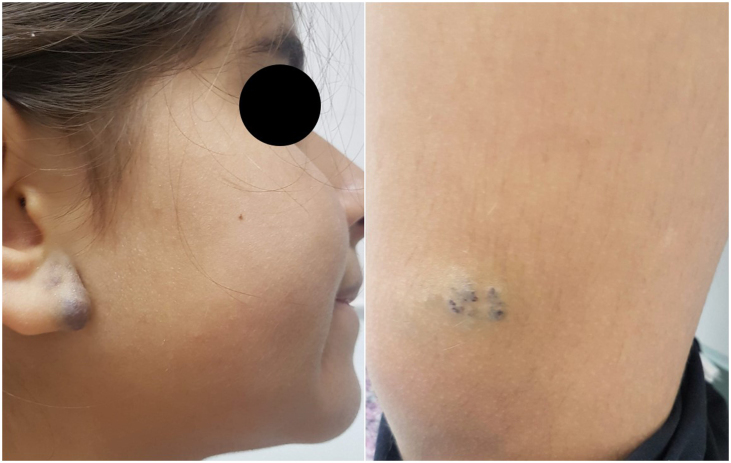
BRBNS. Violaceous papule on the right earlobe and violaceous lesion on the left thigh, with slight swelling on palpation.

**Figure 2:**
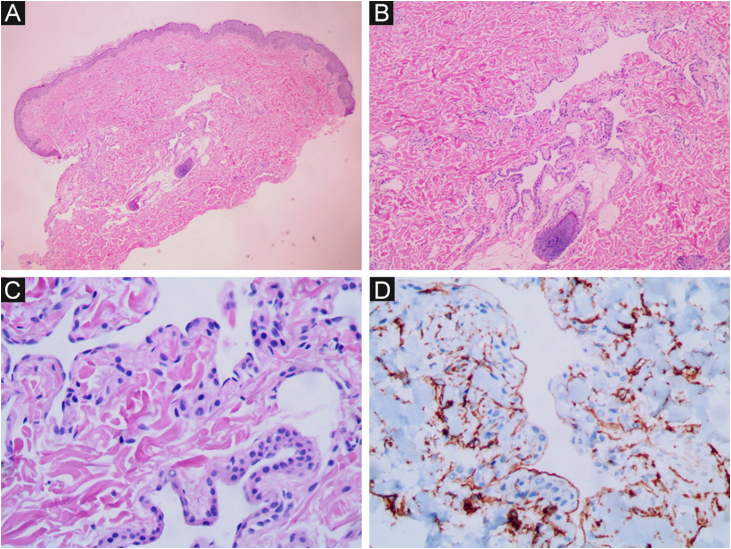
BRBNS. Dilated capillaries lined by a thin layer of endothelial cells. (A) Hematoxylin & eosin (×40). (B) Hematoxylin & eosin, (×100). (C) Hematoxylin & eosin (×400). (D) Immunohistochemistry positive for CD34, showing the vascular endothelium (×400).

An 11-year-old female had a history of bluish lesions on the right earlobe and left thigh since birth. The parents reported progressive increase in lesion size over the years and the appearance of new similar lesions. The clinical examination showed a violaceous lesion on the left thigh with slight swelling on palpation, measuring 4×1 cm, a violaceous papule on the right earlobe measuring 1.5×1 cm, discrete violaceous spots on the lumbar region and a small violaceous spot on the right shoulder, measuring 0.5 cm (Fig. 1). The patient had been diagnosed with polycystic kidney disease and was being followed by a nephrologist. The Doppler echocardiogram of the left thigh showed a hypervascular lesion with predominantly venous flow and no signs of an arteriovenous fistula. The cranial angiotomography showed ectasia of cortical veins in both parietal regions. Histopathology of the lesion in the dorsal region showed dilated dermal capillaries positive for CD34 on immunohistochemistry. These findings were compatible with blue rubber bleb nevus syndrome (Fig. 2). Colonoscopy and endoscopy of the upper digestive tract showed no alterations.

### Case 2

**Figure 3:**
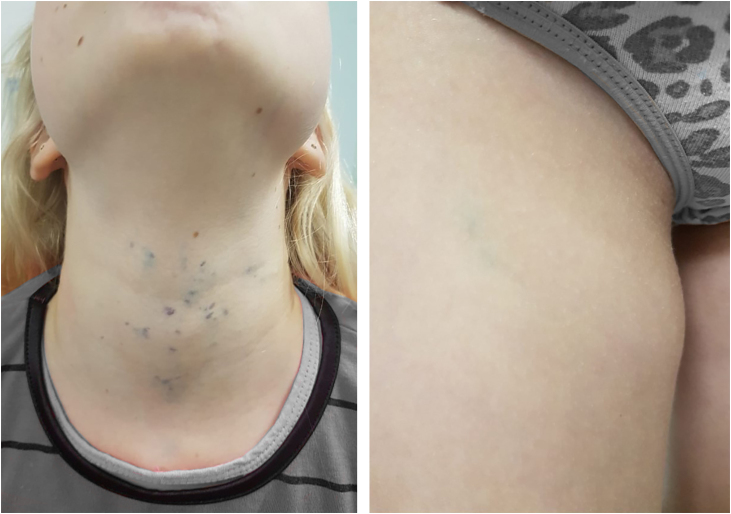
Glomangiomas. Multiple vascular lesions in the cervical region and a single palpable lesion on the right thigh.

**Figure 4:**
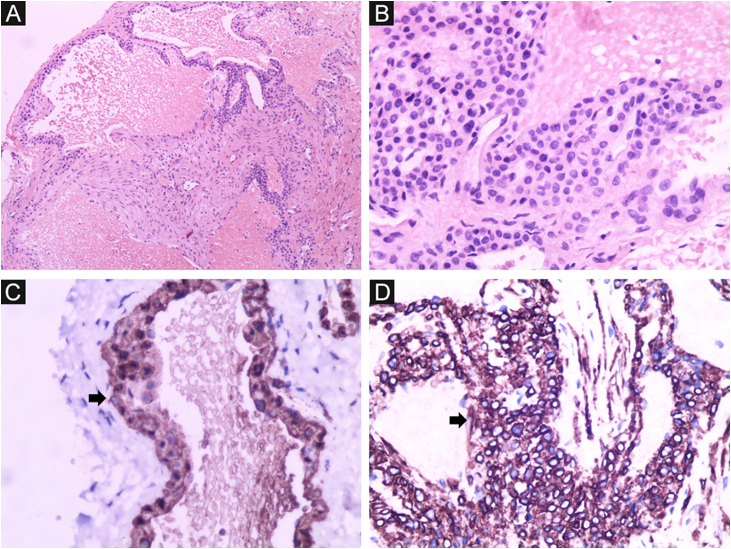
Glomangioma. Dilated vascular spaces lined by glomus cells. (A) Hematoxylin & eosin (×100). (B) Hematoxylin & eosin (×400); (C) Immunohistochemistry positive for smooth muscle actin (×400). (D) Immunohistochemistry positive for caldesmon ×400).

A 13-year-old healthy female was referred to the Dermatology Service for evaluation of a multifocal vascular lesion in the cervical region, present since birth. She reported the recent appearance of new lesions on her face and right thigh (Fig. 3). The Computed tomography angiography (CTA) of the cervical region showed the presence of a group of small vessels with subcutaneous, semilunar distribution in the lateral cervical region, in addition to large vessels with normal diameters and trajectories. The histological study of the lesion on the right thigh showed a poorly circumscribed vascular proliferation in the skin, dilated vascular spaces, and the presence of glomus cells lining the endothelium. Immunohistochemistry was positive for smooth muscle actin, caldesmon and negative for CD34, desmin and S100 (Fig. 4). The histopathological and immunohistochemical findings were compatible with the diagnosis of glomangioma.

## Discussion

BRBNS, also known as Bean syndrome, is a rare condition, in which multiple vascular malformations usually arise in the skin and digestive tract, and which can also affect other organs such as the heart, liver, spleen, eyes, central nervous system, lung, bladder, thyroid and parotids. The lesions appear more frequently in the skin and small intestine and may lead to digestive bleeding and iron deficiency anemia. The lesions may be present at birth but tend to increase in size and number throughout life.^1^, ^4^, ^5^ Glomangiomas originate from the glomus bodies in the venous system. Glomus cells are modified smooth muscle cells that are important in regulating body temperature.^6^ Glomangiomas usually present as multiple lesions that may be present at birth or appear later, during childhood. The lesions do not usually affect the subungual region and may affect systemic organs. Before the anatomopathological evaluation, BRBNS is usually the most frequently considered diagnosis.^3^ In addition, it is important to consider other differential diagnoses for multiple glomangioma, such as hemangiomas, venous malformations, and Maffucci syndrome.^1^

Both primary lesions are described as bluish papules with vascular appearance and varying sizes. Both types of lesions tend to be painless, but painful lesions are not uncommon. Glomangiomas can be painful during pregnancy and menstrual periods.^4^ Moreover, both lesions show a wide cutaneous distribution, potentially affecting the face, trunk, or extremities.^3^, ^6^, ^7^ An important consideration is that multiple glomangiomas primarily affect the skin, whereas the involvement of the skin and the gastrointestinal tract is very common in BRBNS.^8^

Autosomal dominant inheritance has also been demonstrated in both diseases.^1^, ^6^ In BRDNS, it is linked to somatic mutations in the TEK gene encoding the TIE2 protein, a tyrosine kinase-like membrane receptor for angiopoietins (vascular growth factors) present in endothelial cells. Multifocal malformations such as BRBN are predominantly caused by two somatic mutations in the same TEK gene allele.^1^ In glomangiomas, an autosomal dominant pattern with incomplete penetrance and variable expression caused by a mutation in the glomulin gene (GLMN) located on chromosome 1p21-p22 has been described.^6^, ^9^

On histopathology, dilated vascular spaces and the presence of glomus cells lining the endothelium are unique to glomangiomas, together with positive immunohistochemistry for SMA (marker of actin-related protein present in smooth muscle), caldesmon (marker of actin- and calmodulin-related protein present in smooth muscle) and myosin (marker of protein present in muscles), which confirms the muscular nature of these cells. These immunohistochemical markers are negative in BRBNS. In the latter, irregular dilated capillary spaces surrounded by a thin layer of endothelial cells (which are CD34-positive) can be identified in the dermis or subcutaneous adipose tissue.^6^, ^7^

The diagnosis of BRBN is confirmed by histopathological and immunohistochemical examination and a wide range of therapies have been described, from watchful waiting to surgical excision, laser therapy, sclerotherapy of lesions and systemic sirolimus. Treatment will vary according to the symptoms, organ involvement, and development of complications over the course of the disease, taking into account that patients with mild gastrointestinal bleeding can be managed conservatively with iron supplements and transfusions. On the other hand, patients showing greater severity are advised to start systemic treatment with sirolimus. Sirolimus acts by inhibiting angiogenesis and has shown good results.^2^, ^7^, ^10^ Treatments described for glomangioma, on the other hand, involve surgical excision, laser therapy and sclerotherapy of the lesions or watchful waiting.^7^, ^8^

The clinical similarities between these two conditions in the absence of histological study can lead to an incorrect diagnosis and, therefore, to inadequate treatment.^3^ It should be noted that a multidisciplinary therapeutic approach is recommended in patients with both diagnoses.

## Financial support

None declared.

## Authors’ contributions

Elsa Stella Mosquera-Belalcazar: Drafting and editing of the manuscript.

Aline Alves Domingues: Review of the literature and drafting of the manuscript.

Alessandra Coppinni: Review of the literature and drafting of the manuscript.

Laura Luzzatto: Effective participation in data analysis; approval of the final version of the manuscript.

Ana Elisa Kiszewski: Design and planning of the study; critical review of the manuscript and approval of the final version of the manuscript.

## Conflicts of interest

None declared.
